# An alternative dietary variety score reflects nutrient adequacy across different life stages in Japanese women

**DOI:** 10.3389/fnut.2026.1848503

**Published:** 2026-06-11

**Authors:** Yuko Tateishi, Hidehiro Nakamura, Ryoko Tajima, Naoki Hayashi, Kentaro Murakami, Hitomi Okubo

**Affiliations:** 1 Institute of Food Sciences and Technologies, Ajinomoto Co., Inc., Tokyo, Kanagawa, Japan; 2 Department of Nutritional Epidemiology and Behavioural Nutrition, Graduate School of Medicine, The University of Tokyo, Tokyo, Japan; 3 Research Institute for Bioscience Products & Fine Chemicals, Ajinomoto Co., Inc., Tokyo, Kanagawa, Japan; 4 Department of Social and Preventive Epidemiology, School of Public Health, The University of Tokyo, Tokyo, Japan

**Keywords:** dietary variety score, Japan, nutrient adequacy, nutrient intake, women

## Abstract

**Background:**

The Dietary Variety Score (DVS) is a simple indicator of dietary variety widely used in Japan, particularly among older adults. However, its applicability to younger populations remains uncertain, and some components—most notably “fats and oils”—may not fully reflect current nutritional priorities, including the promotion of whole grains for the prevention of noncommunicable diseases. We developed an Alternative Dietary Variety Score (ADVS) and examined whether the original and alternative scores capture food group and nutrient intake, as well as nutrient adequacy, among women across different life stages.

**Methods:**

The cross-sectional study included 4,227 young (mean age 18.2 years), 3,562 middle-aged (47.8 years), and 1,655 older (74.6 years) Japanese women. Dietary intake was assessed using a comprehensive Diet History Questionnaire for young and middle-aged women and a brief-type Diet History Questionnaire for older women. The DVS was calculated based on the weekly consumption frequency of ten food groups, assigning one point for each food group consumed at least once per day. The ADVS retained the original scoring framework but replaced “fats and oils” with “whole grains.” Associations with food groups and nutrient intake were examined using Spearman’s rank correlation coefficients, and nutrient adequacy was evaluated based on the Dietary Reference Intakes for Japanese.

**Results:**

Both DVS and ADVS were positively associated with the intake of vegetables, fruits, fish and shellfish, eggs, soy products, dairy, seaweeds, and potatoes. The ADVS additionally reflected whole-grain consumption, while correlations with fats and oils were attenuated. Both scores were positively correlated with intakes of protein, dietary fiber, vitamins, and minerals across age groups. Higher scores were associated with a lower prevalence of inadequate nutrient intake across all age groups. The ADVS showed somewhat higher correlations with dietary fiber, vitamins B_1_, B_2_ and B_6_, and calcium, iron, and magnesium.

**Conclusion:**

Both the DVS and ADVS are useful proxies of nutrient adequacy among young, middle-aged, and older Japanese women. The ADVS additionally reflected whole-grain consumption and showed a different pattern of correlations with several nutrients, suggesting that incorporating whole grains into a dietary variety index may provide an alternative perspective on dietary variety while maintaining simplicity.

## 1 Introduction

Assessing overall diet quality is essential for understanding the relationship between diet and health outcomes and for informing public health nutrition strategies. Although detailed dietary assessment methods, such as dietary records and 24-h recalls, provide comprehensive information, their use is often limited by substantial time and resource demands. Consequently, dietary diversity indicators (DDIs), which capture the number of different food items or food groups consumed over a specified period, have been widely adopted as simple and feasible tools for evaluating overall diet quality in epidemiological research (1).

Numerous DDIs have been proposed and shown to serve as useful proxies for nutrient adequacy and health outcomes, particularly in populations at risk of undernutrition (1). However, most existing DDIs have been developed and applied in settings where insufficient intake across multiple food groups is the primary concern, such as in low-income countries (2) or among older populations (3). Their applicability to other populations remains unclear. Recently, evidence suggests that inadequate nutrient intake may be more prevalent among younger adults than older adults in high-income countries, including Japan (4–6). These findings highlight the need to re-evaluate whether existing DDIs appropriately capture diet quality across younger adults in contemporary food environments.

In Japan, the Dietary Variety Score (DVS) is a widely used DDI originally developed for community-dwelling older adults (7). The DVS assesses habitual dietary variety based on the weekly consumption frequency of ten food groups—meat; fish and shellfish; eggs and egg products; soybeans and soybean products; milk and dairy products; green and yellow vegetables; seaweed; potatoes; fruits; and fats and oils—assigning one point for each food group consumed almost every day (7). The DVS has been shown to be effective in identifying poor dietary variety among older adults, with higher scores consistently associated with reduced risks of frailty (8–10), sarcopenia (11, 12), and depressive symptoms (13). A modified version of the DVS has also been proposed by expanding food group categories (14). However, because the DVS was designed to detect general nutrient insufficiency in later life, its ability to reflect diet quality in younger populations—where nutritional challenges may arise from dietary imbalance rather than simple undernutrition—remains uncertain.

Accumulating evidence from recent nutritional epidemiological studies underscores the need to reconsider the components of the DVS, particularly with regard to carbohydrate quality. Whole grains, which better reflect carbohydrate quality and provide fiber and essential micronutrients, are now recognized as a key component of healthy dietary patterns and have been consistently associated with a lower risk of major chronic diseases (15, 16). In Japan, low intake of whole grains has been identified as a leading dietary risk factor for noncommunicable diseases (17), yet consumption remains particularly low among younger populations. In contrast, “fats and oils,” one of the original DVS components, may be less suitable as an indicator of dietary variety in contemporary diets, as they are often consumed as invisible ingredients in composite or processed foods.

We therefore developed an Alternative Dietary Variety Score (ADVS) by replacing the “fats and oils” component with “whole grains” while retaining the original scoring framework. Using data from a cross-sectional study of young, middle-aged, and older Japanese women, this study aimed to examine whether the original DVS and ADVS are associated with food group and nutrient intakes, as well as nutrient adequacy based on the Dietary Reference Intakes for Japanese (DRIs), within each age group. We hypothesized that both scores would be associated with nutrient adequacy within each age group, and that the ADVS may show a different pattern of correlations with dietary fiber and micronutrient intake.

## 2 Materials and methods

### 2.1 Survey design

The present cross-sectional study used data from the Three-Generation Study of Women on Diets and Health, a self-administered questionnaire survey conducted in northern and western Japan in 2011 and in eastern Japan in 2012. Full details of the study design and procedures have been published previously (18). Briefly, a total of 7,016 dietetic students from 85 academic institutions across 35 of 47 prefectures were invited to complete two questionnaires on dietary habits and lifestyle during orientation or the first lecture for freshmen in April 2011 or 2012. Students were also asked to distribute similar questionnaires to their mothers and either their grandmothers or, if unavailable, another female acquaintance aged 65–89 years. Participants were therefore recruited through family connections (e.g., students, their mothers, and grandmothers), and some participants may have shared household environments. Participants who agreed to take part provided written informed consent and completed the questionnaire, which was returned in a sealed envelope via the students. The study office checked all questionnaires for completeness and logical consistency; participants with missing or inconsistent responses were asked to resubmit them. The response rates were 70.3% for students, 57.6% for mothers, and 33.2% for the grandmother generation. This study was conducted in accordance with the Declaration of Helsinki and approved by the Research Ethics Committees of the University of Tokyo Faculty of Medicine and Ajinomoto Co., Inc. Written informed consent was obtained from all participants; for those under 20 years of age, consent was also obtained from a parent or guardian.

### 2.2 Analytic sample

In this study, university students were classified as young women, mothers as middle-aged women, and the grandmother generation as older women. The selection process for the analytic sample is shown in Supplementary Figure 1.

For the young women, 4,956 participants with available dietary survey data were screened. Exclusions included males (*n* = 280); implausible energy intake (<850 or ≥3,375 kcal/day for ages 18–29 years, based on the Estimated Energy Requirements (EERs) defined in the DRIs for Japanese, 2025; *n* = 170); residence in regions affected by the Great East Japan Earthquake (*n* = 45); questionnaire completion after May 19, to avoid potential influence from nutrition classes (*n* = 54); age ≥30 years (*n* = 37); and missing intake data for food groups required to calculate dietary diversity scores (*n* = 181). After removal of duplicate entries, 4,227 young women were included in the final analysis.

For the middle-aged women, 4,074 participants with dietary data were screened. Exclusion criteria included implausible energy intake (<875 or ≥3,525 kcal/day for ages 30–49 years; <850 or ≥3,375 kcal/day for ages 50–64 years; *n* = 77); residence in earthquake-affected regions (*n* = 63); age ≥65 years or unknown age (*n* = 11); and missing intake data for relevant food groups (*n* = 363). After removal of duplicates, 3,562 middle-aged women were included.

For the older women, 2,366 participants with available dietary data were screened. Exclusion criteria included males (*n* = 4); implausible energy intake (<825 or ≥3,075 kcal/day for ages 65–74 years; <725 or ≥2,625 kcal/day for ages ≥75 years; *n* = 114); residence in earthquake-affected regions (*n* = 47); age <65 years or unknown age (*n* = 79); residence outside Japan (*n* = 1); and missing intake data for relevant food groups (*n* = 477). After removing duplicates, 1,655 older women were included in the final analysis.

### 2.3 Dietary assessment

Dietary habits during the preceding month were assessed using a validated comprehensive Diet History Questionnaire (DHQ) for young and middle-aged women and a validated brief-type Diet History Questionnaire (BDHQ) for older women (19–21). The DHQ and BDHQ are structured, self-administered questionnaires that assess the consumption frequency of foods commonly consumed in Japan; the DHQ additionally collects information on portion sizes, general dietary behaviors, and usual cooking methods. Daily intake of foods (151 items for DHQ, 58 items for BDHQ), energy, and nutrients was estimated using an *ad hoc* computer algorithm based on the Standard Tables of Food Composition in Japan. The relative validity of the DHQ and BDHQ has been evaluated previously among 92 women aged 31–69 years using 16-day weighed dietary records as the reference method (20, 21). For the DHQ, the median Spearman correlation coefficient for food group intake was 0.43 (range, −0.09 to 0.77), and the median Pearson correlation coefficient for nutrient intake was 0.57 (range, 0.27 to 0.87). For the BDHQ, the corresponding median values were 0.44 (range, 0.14 to 0.82) for food groups and 0.54 (range, 0.34 to 0.87) for nutrients, based on comparisons with dietary records (20, 21). To reduce the impact of misreporting, energy-adjusted values were calculated using the density method (i.e., percentage of energy for energy-providing nutrients and amount per 1,000 kcal for other nutrients and foods) (22).

### 2.4 Assessment of dietary variety: DVS and ADVS

Dietary variety was assessed using two complementary scores, the DVS and ADVS, each ranging from 0 to 10 points. The DVS, originally developed by Kumagai et al. (7), is a simple and widely used indicator of dietary variety in Japan, particularly among older adults. It comprises ten food groups: meat, fish and shellfish, soybeans and soybean products, milk, green and yellow vegetables, seaweed, potatoes, fruits, eggs, and fats and oils. In this study, “milk” was operationalized as “milk and dairy products” to align with the classification used in our dietary questionnaires. Although the DVS provides a practical measure of dietary variety, some components—most notably fats and oils—may not fully reflect current nutritional priorities, such as the promotion of whole grains for the prevention of noncommunicable diseases (17). In this study, the ADVS was created by replacing the “fats and oils” component with “whole grains,” defined based on questionnaire items on the consumption of rice other than white rice, including brown rice, germinated rice, and rice mixed with barley or other grains. This approach was intended to ensure comparability of whole-grain classification across both questionnaire types and age groups, although it does not quantify absolute intake levels.

For both scores, 1 point was assigned if a food group was consumed daily; otherwise, 0 points were assigned. An exception was made for “fats and oils” in the DVS, which scored 1 point if intake met or exceeded the generation-specific median, reflecting its typical consumption as an invisible ingredient in composite dishes. Specific food items used for scoring are listed in Supplementary Table 1. Weekly consumption frequencies for each food group were estimated from DHQ responses for young and middle-aged women and from BDHQ responses for older women. Standard frequency conversions were applied: “2 or more times per day” = 17.5 times/week, “once per day” = 7 times/week, “4–6 times per week” = 5 times/week, “2–3 times per week” = 2.5 times/week, “once a week” = 1 time/week, “2–3 times a month” or “less than once a week” = 0.6 times/week; “once a month” = 0.2 times/week; and “none” = 0 times/week. Because fruit and vegetable juice—categorized as “green and yellow vegetables” or “fruits”—was assessed using different frequency options than other food groups, and fats/oils were assessed using the dietary history method, these were treated separately (see Supplementary Table 2 for detailed conversions).

For the ADVS, scoring of whole grains was harmonized across questionnaires and generations. In the DHQ, participants who answered “yes” to the question “Did you regularly consume grains other than white rice, such as barley, germinated rice, partially milled rice (50% or 70% polished), or brown rice?” were assigned 1 point, whereas those who answered “no” were assigned 0 points. In the BDHQ, participants who answered “always,” “sometimes,” or “rarely” to the question “Do you eat brown rice or germinated rice, or mix barley or other grains with rice?” were assigned 1 point, whereas those who answered “no” were assigned 0 points. This approach was intended to improve comparability of whole-grain–type food inclusion across questionnaire types and age groups while maintaining the interpretability and practical simplicity of the original DVS framework.

### 2.5 Assessment of nutritional adequacy

Nutrient adequacy was evaluated by comparing nutrient intakes with age- and sex-specific reference values in the DRIs for Japanese, 2025 (23) or, when appropriate, with World Health Organization recommendations (24). Because self-administered dietary questionnaires, such as the DHQ and BDHQ, are subject to measurement error, nutrient intakes were energy-adjusted using the density method to facilitate comparison with the DRIs. EERs, based on age- and sex-specific reference values for a low physical activity level, reflecting the predominantly sedentary lifestyle of this population (25), were used as reference values for evaluating nutrient adequacy. Energy-adjusted intakes were calculated as:

Energy-adjusted nutrient intake (amount/day) = crude intake of each nutrient (amount/day) × EER (kcal/day)/observed energy intake (kcal/day).

The Estimated Average Requirement (EAR) and Tentative Dietary Goal for preventing lifestyle-related diseases (DG) were used to classify intake adequacy. The EAR represents “the intake estimated to meet the requirement of 50% of individuals in a given age- and sex-specific group,” whereas the DG defines “the intake range recommended to reduce the risk of lifestyle-related diseases in the population” (23). For nutrients with EAR—protein, vitamin A (retinol equivalent), vitamin B_1_, vitamin B_2_, niacin (niacin equivalent), vitamin B_6_, folate, vitamin C, calcium, magnesium, zinc, copper, and iron—participants with energy-adjusted intakes below the EAR were classified as having inadequate intake (23). Iron was treated separately due to highly skewed requirements among menstruating women; assuming a 15% absorption rate, intakes below 9.3 mg/day were considered inadequate for women aged 20–49 years (24, 26). For nutrients with DGs—protein, total fat, saturated fatty acids (SFAs), carbohydrate, dietary fiber, sodium, and potassium—participants with energy-adjusted intakes that fell outside the recommended range were classified as inadequate. Reference values and prevalence of inadequate intakes in this population are presented in Supplementary Table 3.

### 2.6 Assessment of other variables

Other variables used were based on participants’ self-reported information. Age at the time of the survey was calculated from the participants’ date of birth. Body mass index was calculated as body weight (kg) divided by the square of body height (m^2^).

### 2.7 Statistical analysis

Descriptive statistics for participant characteristics were presented as means and standard deviations for continuous variables and as counts and percentages for categorical variables, separately for each age group. For each food group, the number and proportion of participants reporting daily consumption (based on the data used for DVS and ADVS calculations) were determined. Spearman’s rank correlation coefficients were calculated to examine the associations of the DVS and ADVS with food group and nutrient intakes. Thirteen food groups were analyzed, including the 11 groups used in the DVS/ADVS calculation, as well as cereals (rice, noodles, and bread) and confectioneries (rice crackers; snack foods; Japanese sweets; cakes; cookies; chocolate; candy/caramel/gum; jelly; and donuts). Participants were categorized into three groups based on their DVS and ADVS scores (0–10 points): low (0–3), medium (4–6), and high (7–10). For each age group and score category, the proportion of participants with inadequate nutrient intake—defined according to EAR or DG after energy adjustment—was calculated and compared across the three score groups. Additionally, overall nutritional inadequacy was evaluated by summing the number of nutrients not meeting the DRIs (13 nutrients with EAR and 7 nutrients with DG), yielding a total score ranging from 0 to 20. All analyses were performed using R software (version 4.0.5), and a *p*-value of less than 0.05 was considered statistically significant.

## 3 Results

### 3.1 Subject characteristics and dietary variety scores

The characteristics of the study participants are shown in Table 1. The mean ages were 18.2 ± 0.9 years for young women, 47.8 ± 4.1 years for middle-aged women, and 74.7 ± 5.0 years for older women. Mean DVS values were 2.9 ± 1.9, 3.6 ± 2.0 and 4.4 ± 2.0 for young, middle-aged, and older women, respectively. The corresponding mean ADVS values were 2.5 ± 1.7, 3.2 ± 1.9, and 4.3 ± 1.9. Both scores showed a slight increase with age. DVS and ADVS were strongly correlated within each age group, with Pearson’s correlation coefficients of 0.95, 0.95, and 0.94 for young, middle-aged, and older women, respectively (all *p* < 0.001). The distributions of total scores were sufficiently wide to detect meaningful differences across all age groups, with similar median and percentile patterns (Supplementary Table 4). The proportions of women consuming each food component of DVS and ADVS at least once per day are presented in Supplementary Tables 5, 6. For all food groups except meat and eggs, the proportion increased with age. Across all age groups, higher DVS and ADVS were consistently associated with greater consumption of all food groups.

**Table 1:** Characteristics of the study participants.

Characteristic	Young	Middle-aged	Older
*N*	4,227	3,562	1,655
Age (years)	18.2 (0.9)	47.8 (4.1)	74.7 (5.0)
Body height (cm)	157.8 (5.3)	157.2 (5.1)	150.3 (5.6)
Body weight (kg)	51.9 (7.8)	54.5 (8.1)	51.5 (8.0)
Body mass index (kg/m^2^)	20.8 (2.8)	22.0 (3.1)	22.8 (3.3)
Energy intake (kcal/day)	1,711 (461)	1,822 (454)	1,709 (439)
Dietary variety score (0–10)	2.9 (1.9)	3.6 (2.0)	4.4 (2.0)
Alternative dietary variety score (0–10)	2.5 (1.7)	3.2 (1.9)	4.3 (1.9)

Values are mean (standard deviation).

### 3.2 Correlations of DVS and ADVS with food group intake

Table 2 presents the Spearman correlation coefficients between DVS/ADVS and intake of 13 food groups. Overall, both scores showed similar correlation patterns, while correlations with confectioneries were negligible. Positive correlations were observed for fish and shellfish, soybean products, dairy products, green and yellow vegetables, seaweeds, potatoes, fruits, and eggs. In contrast, negative correlations were observed for rice, bread, and noodles. Notably, ADVS showed moderate positive correlations with whole grains, reflecting its inclusion as a component, and weaker correlations with fats and oils compared with DVS.

**Table 2:** Spearman correlation coefficients of dietary variety score (DVS) and alternative dietary variety score (ADVS) with food group intake across age groups.

Food group	Young		Middle-aged		Older	
	DVS	ADVS	DVS	ADVS	DVS	ADVS
Meat	0.37	0.32	0.24	0.19	0.23	0.17
Fish and shellfish	0.30	0.29	0.26	0.27	0.33	0.34
Soybeans and soybean products	0.34	0.37	0.38	0.42	0.35	0.40
Milk and dairy products	0.22	0.26	0.27	0.31	0.24	0.28
Green and yellow vegetables	0.45	0.48	0.40	0.43	0.22	0.24
Seaweeds	0.27	0.28	0.28	0.31	0.32	0.34
Potatoes	0.23	0.23	0.24	0.25	0.25	0.26
Fruits	0.26	0.28	0.32	0.36	0.36	0.39
Eggs	0.24	0.25	0.24	0.22	0.26	0.26
Fats and oils	0.36	0.15	0.28	0.10	0.26	0.06
Rice, bread, and noodles	−0.54	−0.49	−0.51	−0.44	−0.53	−0.48
Confectioneries	0.02	0.01	0.06	0.04	0.04	0.01
Whole grains	0.10	0.30	0.08	0.27	0.06	0.30

Values are spearman correlation coefficients. Except for confectionaries, all other food groups are significantly correlated with both DVS and ADVS (*p* < 0.01). DVS, dietary variety score; ADVS, alternative dietary variety score.

### 3.3 Correlations of DVS and ADVS with nutrient intake

Table 3 shows the correlations between DVS/ADVS and nutrient intake. Across all age groups, both scores demonstrated broadly similar patterns. Positive correlations were observed for protein, fat, niacin, vitamins B_1_, B_2_, and B_6_, calcium, potassium, iron, and zinc, whereas carbohydrate intake was negatively correlated with both scores. Compared with DVS, ADVS tended to show lower correlations with total fat and SFAs and higher correlations with dietary fiber, B vitamins, and several minerals, including calcium, potassium, magnesium, iron, and zinc.

**Table 3:** Spearman correlation coefficients of dietary variety score (DVS) and alternative dietary variety score (ADVS) with nutrient intake across age groups.

Nutrients	Young		Middle-aged		Older	
Unit:/1,000 kcal	DVS	ADVS	DVS	ADVS	DVS	ADVS
Protein (% energy)	0.51	0.55	0.47	0.51	0.48	0.51
Fat (% energy)	0.52	0.41	0.44	0.33	0.46	0.35
Carbohydrate (% energy)	−0.55	−0.46	−0.41	−0.32	−0.47	−0.41
Dietary fiber (g)	0.32	0.40	0.33	0.41	0.34	0.40
Vitamin D (μg)	0.36	0.37	0.34	0.36	0.33	0.36
Retinol (μgRAE)	0.45	0.47	0.39	0.42	0.29	0.31
Niacin (mg NE)	0.49	0.55	0.33	0.38	0.43	0.45
Vitamin B_1_ (mg)	0.51	0.57	0.38	0.44	0.50	0.53
Vitamin B_2_ (mg)	0.39	0.42	0.38	0.42	0.42	0.47
Vitamin C (mg)	0.36	0.38	0.36	0.39	0.32	0.35
Vitamin B_6_ (mg)	0.54	0.61	0.46	0.53	0.45	0.49
Folate (μg)	0.36	0.40	0.36	0.41	0.30	0.33
Calcium (mg)	0.43	0.48	0.45	0.50	0.43	0.49
Potassium (mg)	0.55	0.61	0.46	0.52	0.44	0.49
Iron (mg)	0.45	0.50	0.43	0.50	0.39	0.45
Zinc (mg)	0.41	0.47	0.37	0.45	0.39	0.43
Magnesium (mg)	0.44	0.55	0.37	0.47	0.44	0.51
Copper (mg)	0.14	0.23	0.22	0.32	0.25	0.34
Sodium (mg)	0.29	0.27	0.24	0.22	0.08	0.08
Saturated fatty acids (% energy)	0.39	0.35	0.36	0.31	0.36	0.32

All nutrient intakes significantly correlated with both DVS and ADVS (*p* < 0.01). DVS, Dietary Variety Score; ADVS, Alternative Dietary Variety Score; RAE, retinol activity equivalent; NE, niacin equivalent.

### 3.4 Associations of DVS and ADVS with nutrient adequacy

**Figure 1:**
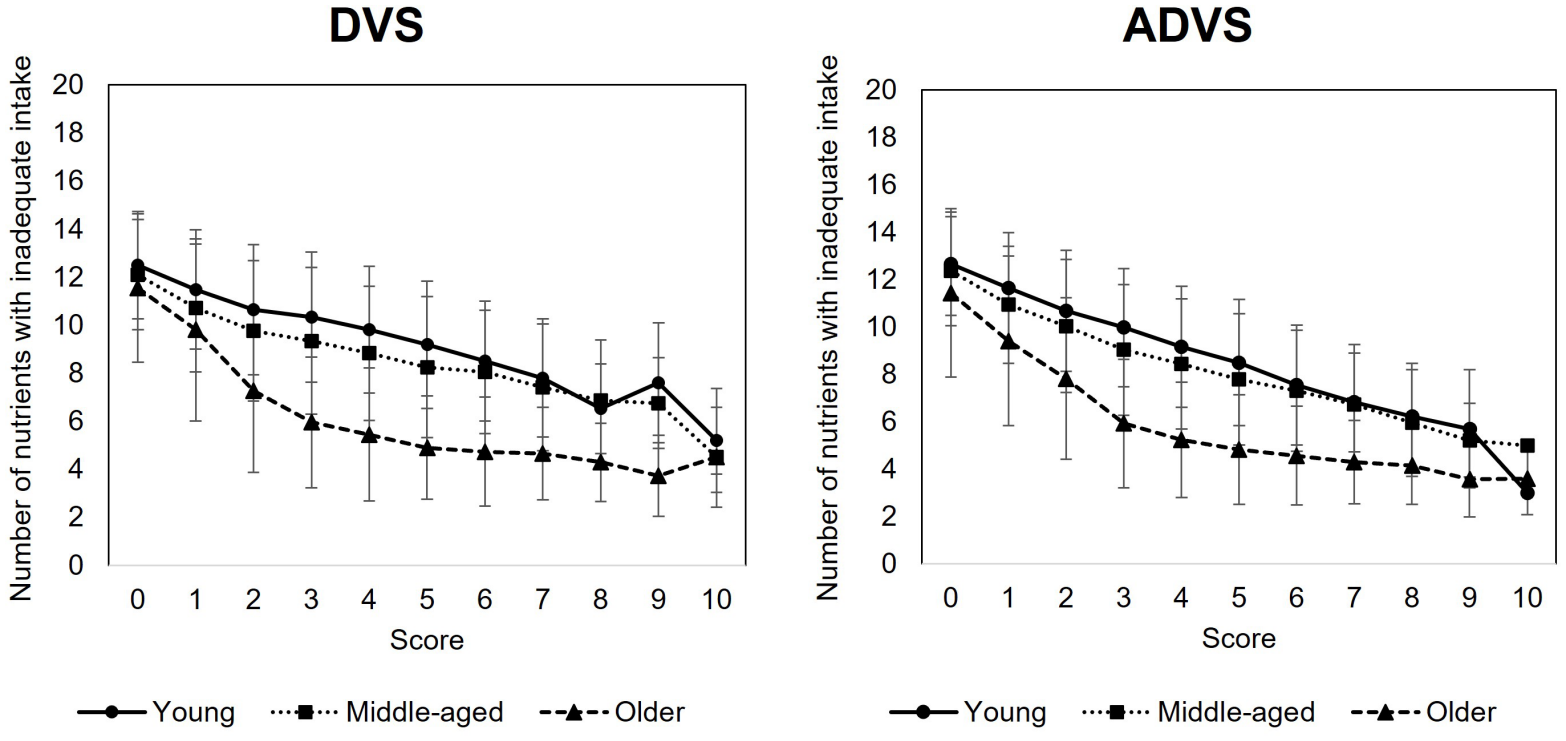
Number of nutrients with inadequate intake according to dietary variety score (DVS) and alternative dietary variety score (ADVS) categories across age groups.

Table 4 presents the prevalence of inadequate nutrient intake across low (0–3), medium (4–6), and high (≥7) categories of DVS and ADVS. In all age groups, the prevalence of inadequate intake for most nutrients decreased as scores increased, except for carbohydrates, sodium, and SFAs. For several water-soluble vitamins (B_2_, B_6_, and C) and minerals (calcium, iron, and magnesium), inadequacy was highest in the low-score group and lowest in the high-score group. This gradient was generally more pronounced for ADVS than DVS, particularly among younger and middle-aged women. In contrast, excessive sodium intake remained consistently high (approximately 80–90%) across all score categories, and higher scores were associated with a greater prevalence of excessive SFAs intake. Carbohydrate intake did not follow the general pattern observed for other nutrients. Figure 1 shows the associations of DVS and ADVS with overall nutritional inadequacy, defined as the number of nutrients not meeting the DRIs. Higher scores were associated with fewer inadequacies across all age groups, with a steeper decline among older women. The reduction was slightly greater for ADVS than for DVS.

**Table 4:** Prevalence of inadequate nutrient intake by low, medium, and high dietary variety score (DVS) and alternative dietary variety score (ADVS) across age groups.

Participants (N) and Nutrients	DRI category	Young						Middle-aged						Older					
		DVS			ADVS			DVS			ADVS			DVS			ADVS		
		Score 0–3	Score 4–6	Score 7–10	Score 0–3	Score 4–6	Score 7–10	Score 0–3	Score 4–6	Score 7–10	Score 0–3	Score 4–6	Score 7–10	Score 0–3	Score 4–6	Score 7–10	Score 0–3	Score 4–6	Score 7–10
*N*		2,779	1,290	158	3,152	983	92	1,797	1,487	278	2,096	1,296	170	567	825	263	611	839	205
Nutrients with EAR																			
Protein (g)	<EAR	5	1	0	4	0	0	2	0	0	2	0	0	2	0	0	2	0	0
Retinol (μgRAE)	<EAR	73	52	25	72	47	15	72	55	42	71	52	38	34	18	5	34	16	4
Niacin (mgNE)	<EAR	0	0	0	0	0	0	0	0	0	0	0	0	0	0	0	0	0	0
Vitamin B_1_ (mg)	<EAR	28	4	0	25	3	0	16	3	1	15	3	1	17	3	0	18	2	0
Vitamin B_2_ (mg)	<EAR	40	13	3	38	10	1	29	8	3	27	7	4	18	3	0	18	3	0
Vitamin C (mg)	<EAR	67	48	20	66	43	15	66	47	24	66	43	19	25	8	3	24	8	1
Vitamin B_6_ (mg)	<EAR	82	51	15	81	40	7	64	34	14	63	28	10	37	12	5	36	11	3
Folate (μg)	<EAR	38	17	1	36	14	0	27	9	3	26	7	2	10	1	0	10	1	0
Calcium (mg)	<EAR	89	74	53	89	68	43	85	69	49	85	65	41	64	39	17	66	35	14
Iron (mg)	<EAR	98	94	87	99	91	82	73	67	62	74	66	56	7	1	0	7	0	0
Magnesium (mg)	<EAR	89	75	41	91	65	24	76	59	37	78	51	25	59	35	16	61	32	13
Zinc (mg)	<EAR	23	9	1	22	6	0	37	19	7	37	15	4	22	10	5	23	9	3
Copper (mg)	<EAR	0	0	0	0	0	0	0	0	0	0	0	0	0	0	0	0	0	0
Nutrients with DG																			
Protein	<DG	64	29	8	62	23	3	57	27	11	55	23	9	51	18	6	51	16	5
(% energy)	>DG	0	1	0	0	1	1	0	0	1	0	1	1	7	17	33	7	17	38
Fat	<DG	9	1	1	8	2	2	9	1	0	8	2	0	29	6	0	26	7	1
(% energy)	>DG	33	68	85	39	63	77	28	58	70	35	57	62	9	21	35	13	21	29
Carbohydrate	<DG	9	34	58	13	33	58	15	33	46	19	32	41	9	22	39	12	22	38
(% energy)	>DG	15	2	1	14	3	2	10	1	1	8	2	0	23	4	0	21	5	1
Fiber (g)	<DG	98	95	82	99	92	77	97	94	91	97	93	86	95	91	88	95	91	84
Potassium (mg)	<DG	98	92	73	98	89	64	94	83	69	94	81	58	75	55	32	77	53	26
Sodium (mg)	≥DG	84	92	94	85	93	92	91	95	97	91	96	99	96	98	99	96	98	98
Saturated fatty acids (% energy)	>DG	61	85	92	64	84	87	57	80	89	61	79	87	25	42	59	28	43	54

Values are percentage (%); DVS, Dietary Variety Score; ADVS, Alternative Dietary Variety Score; EAR, estimated average requirement; RAE, retinol activity equivalent; NE, niacin equivalent; DG, a tentative dietary goal for preventing lifestyle-related disease.

Nutrient intakes were energy-adjusted using the following formula: Energy-adjusted intake = observed intake × Estimated Energy Requirement for low physical activity level (kcal/day)/observed energy intake (kcal/day).

Inadequate nutrient intake was assessed based on the Dietary Reference Intakes for Japanese (2025).

Results for iodine, selenium, and molybdenum are not presented, as their intake could not be calculated using the diet history questionnaire (DHQ) or the brief-type diet history questionnaire (BDHQ).

## 4 Discussion

In this cross-sectional study of Japanese women across different age groups, both the DVS and ADVS were associated with improved overall nutrient adequacy. Higher scores were associated with a lower number of nutrients not meeting the DRIs, suggesting that dietary variety serves as a useful indicator of overall nutritional adequacy in this population. These findings support the value of simple dietary variety indices as practical tools for population-level nutritional assessment. Notably, ADVS showed a different pattern of associations with dietary fiber, several B vitamins, and key minerals. Collectively, these results suggest that incorporating whole grains into a dietary variety index may provide an alternative perspective on dietary variety while maintaining simplicity and usability.

Dietary variety indices have long been used as convenient proxies for nutrient adequacy, especially among older adults. Previous studies in older Japanese populations have shown that higher DVS is associated with greater intakes of protein, vitamins, and minerals along with lower carbohydrate intake (27, 28). The present study extends these observations to younger and middle-aged women, indicating that similar relationships exist across different age groups. Together, these findings reinforce the concept that consuming a wider range of food groups contributes to improved nutrient adequacy throughout adulthood. However, dietary diversity alone does not necessarily reflect overall diet quality, particularly in modern food environments where increased variety may include energy-dense, nutrient-poor foods. Because the original DVS does not include staple foods, it may not fully capture current dietary patterns, especially among younger women, who may restrict staple foods while increasing confectionery intake (29), and who report infrequent consumption of balanced meals consisting of staple foods, main dishes, and side dishes (30). In such contexts, refining the selection of food group components to better reflect balanced diets and reduce nutrient inadequacy may be beneficial.

To address this limitation, we developed the ADVS from the original DVS by replacing the “fats and oils” component with “whole grains,” which are now widely recognized as an essential component of healthy dietary patterns and an important source of dietary fiber, B vitamins, and minerals. A growing body of evidence from both international and Japanese studies has linked higher whole-grain intake with reduced risks of noncommunicable diseases (17). Consistent with this rationale, the ADVS showed different patterns of associations with dietary fiber, B vitamins, and several minerals across all age groups. Although these differences were modest, their consistency suggests that even small, conceptually driven modifications may help dietary variety indices better reflect contemporary dietary patterns, thereby supporting the assessment of diet quality in modern populations. Given the high correlation between the DVS and ADVS and their largely shared components, formal statistical comparisons between their associations were not performed. Accordingly, the results should be interpreted as descriptive comparisons of associations with nutrient intake. Furthermore, the low intake of whole grains observed in this study highlights the relevance of considering whole-grain consumption in dietary variety assessment among Japanese women. The ADVS may be applicable in contexts where dietary patterns have shifted toward increased consumption of refined grains and processed foods, providing an alternative perspective to traditional dietary variety measures.

Although participants included women across three different life stages, the primary objective was not to directly compare age groups but to examine whether the association between dietary variety and nutrient adequacy was consistent within each group. Younger and middle-aged women had lower DVS and ADVS than older women, indicating less diverse diets; however, greater dietary variety was more strongly associated with lower nutrient inadequacy in the younger groups. This suggests that promoting dietary variety may be particularly important earlier in adulthood. Differences in food group consumption across age groups—such as lower intake of potatoes, seaweed, and fruits among younger women—likely reflect generational shifts in dietary habits and preferences influenced by broader social and cultural changes, highlighting the need for age-specific dietary recommendations (31–33).

It should also be noted that both DVS and ADVS primarily capture inadequate intake across food groups and are not designed to assess excessive consumption. In this study, sodium and SFA intake were positively associated with higher scores, possibly reflecting greater use of seasonings and animal-based foods in more varied diets. This may represent a trade-off within traditional Japanese dietary patterns, where increased variety in side dishes and protein sources is accompanied by greater use of sodium-containing seasonings. These findings are consistent with previous reports indicating that diets rich in vegetables and protein sources are often associated with higher sodium consumption (34). Given that excessive sodium intake remains a major public health concern in Japan (35), dietary variety indices should be interpreted alongside complementary measures that specifically address nutrients prone to overconsumption, rather than being considered comprehensive indicators of diet quality.

The strengths of this study include demonstrating the applicability of DVS and ADVS across a broad age range, including younger and middle-aged women, and providing a comprehensive evaluation of these indices in a geographically diverse sample with varied dietary and lifestyle patterns. However, several limitations should be acknowledged. First, both the DVS and ADVS were derived from the DHQ and BDHQ rather than assessed using dedicated dietary variety instruments, and their validity for directly measuring dietary variety has not been formally established. In addition, whole-grain intake may have been imperfectly assessed. The DHQ and BDHQ do not comprehensively capture all whole-grain foods (e.g., oatmeal and bran products), and in the BDHQ, responses such as “always,” “sometimes,” or “rarely” were treated equivalently, which may have overestimated intake among older women. Moreover, the whole-grain component primarily reflects traditional Japanese dietary patterns (e.g., brown rice and barley-mixed rice) and does not quantify absolute intake. Second, the use of different dietary assessment tools for younger and middle-aged women (DHQ) and older women (BDHQ) may have affected the interpretation of differences across age groups. Third, due to the cross-sectional design, causal relationships cannot be inferred, and reverse causation cannot be ruled out. Fourth, participants were recruited through family connections (e.g., students, their mothers, and grandmothers), and dietary behaviors may be correlated within households, which may limit the generalizability. Finally, as the study included only women, the applicability of ADVS to men and other populations requires further investigation.

In conclusion, this study demonstrates that both the DVS and ADVS are useful indicators of nutrient adequacy among Japanese women across different life stages, showing broadly similar associations with food group and nutrient intake. The ADVS showed different patterns of associations with several nutrients, suggesting that incorporating whole grains into a dietary variety index may help better reflect contemporary dietary patterns. These findings highlight the potential for updating simple dietary assessment tools to better capture evolving dietary challenges in modern populations. Further research is needed to assess their applicability in broader populations and to explore how dietary variety indices can be combined with complementary measures to better capture both insufficient and excessive nutrient intake.

## Data Availability

The dataset is not publicly available due to ethical restrictions. Data may be available from the corresponding author upon reasonable request and with approval from the relevant ethics committee. Requests to access the datasets should be directed to Hitomi Okubo, okubo@m.u-tokyo.ac.jp.
