# Supplementary material for: An alternative dietary variety score reflects nutrient adequacy across different life stages in Japanese women
Source: Front Nutr. 2026 Jun 11;13:1848503. doi: 10.3389/fnut.2026.1848503 (PMC13293800; doi:10.3389/fnut.2026.1848503)
Supplement: Supplementary file 1 [file Table_1.docx]

Supplementary Material

# Supplementary Figures


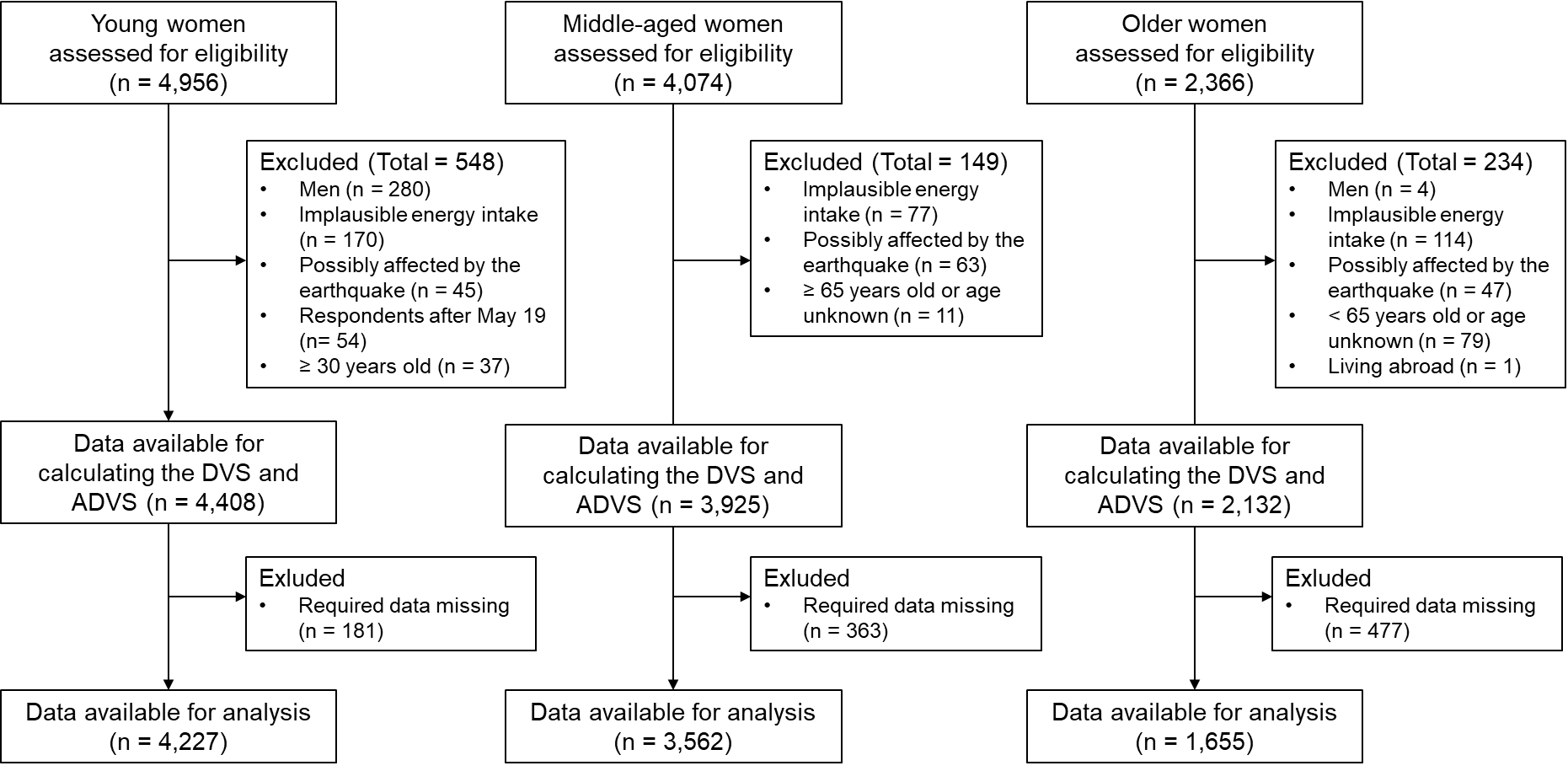


# Supplementary Figure 1. Flowchart of participant selection

DVS; Dietary Variety Score; ADVS, Alternative Dietary Variety Score

# Supplementary Tables

**Supplementary Table 1.** Types of foods used for the calculation of Dietary Variety Score and Alternative Dietary Variety Score

| Food group | Diet History Questionnaire | Brief-type Diet History Questionnaire |
| --- | --- | --- |
| Meat | Ground meat, chicken, pork, beef, liver, ham or sausage, bacon | Chicken, pork/beef, ham/sausage/bacon, liver |
| Fish and shellfish | Dried fish, fish eaten with bones, tuna, eel, white fish, blue-backed fish, red-fleshed fish, processed seafood products, shrimp/crab, squid/octopus, oysters, other shellfish | Squid/octopus, shrimp, fish eaten with bones, canned tuna, dried or salted fish, fatty fish, lean fish |
| Soybeans and soybean products | Tofu, deep-fried tofu, natto, simmered beans, soy milk | Tofu/thick-fried tofu, natto |
| Milk and dairy products | Low-fat milk, regular/high-fat milk, cheese, yogurt, cottage cheese | Low-fat milk, regular/high-fat milk |
| Green and yellow vegetables | Carrot, pumpkin, tomato, bell pepper, broccoli, dark green leafy vegetables, tomato juice, vegetable juice | Dark green leafy vegetables, carrot/pumpkin, tomato, 100% fruit/vegetable juice |
| Seaweeds | Wakame/hijiki, nori (seaweed) | Seaweed |
| Potatoes | Potatoes, other tubers | Tubers |
| Fruits | Mandarin orange, banana, apple, strawberry, grape, peach, pear, persimmon, kiwi fruit, melon, watermelon, 100% fruit juice | Mandarin orange, persimmon/strawberry/kiwi, other fruits |
| Eggs | Egg | Egg |
| Fats/oil | Margarine, mayonnaise, salad dressing, cooking oil | Cooking oil, mayonnaise |
| Whole grains | Barley rice, germinated rice, semipolished rice (50%), semipolished rice (70%), brown rice | Brown rice, germinated rice, barley, mixed grains |

**Supplementary Table 2.** Conversion of Diet History Questionnaire/Brief-type Diet History Questionnaire responses to weekly intake frequency

| Category | Responses to the Diet History Questionnaire or Brief-type Diet History Questionnaire | Weekly intake frequency (converted value) |
| --- | --- | --- |
| Foods (excluding 100% fruit/vegetable juice, fats and oils, and whole grains) | "2 or more times per day" | 2.5 **×** 7 = 17.5 times/week |
|  | "Once per day" | 1 **×** 7 = 7 times/week |
|  | "4–6 times per week" | 5 times/week |
|  | "2–3 times per week" | 2.5 times/week |
|  | "Once a week" | 1 time/week |
|  | "2–3 times a month" or "Less than once a week" | 2.5/30 **×** 7 = 0.6 times/week |
|  | "Once a month" | 1/30 **×** 7 = 0.2 times/week |
|  | "None" | 0 times/week |
| 100% fruit/vegetable juice | "6 or more times per day" | 6.5 **×** 7 = 45.5 times/week |
|  | "4–6 times per day" or "4 or more cups per day" | 4.5 **×** 7 = 31.5 times/week |
|  | "2–3 times per day" | 2.5 **×** 7 = 17.5 times/week |
|  | "Once per day" | 1 **×** 7 = 7 times/week |
|  | "4–6 times per week" | 4.5 times/week |
|  | "2–3 times per week" | 2.5 times/week |
|  | " Once a week" | 1 time/week |
|  | " Less than once a week" | 2.5/30 **×** 7 = 0.6 times/week |
| Fats and oils | **≥**generation-specific median value | 7 times/week |
|  | <generation-specific median value | 0 times/week |
| Whole grains | Responded "yes" on the Diet History Questionnaire or "always," "sometimes," or "rarely" on the Brief-type Diet History Questionnaire to regular whole grain consumption | 7 times/week |
|  | Responded "no" on the Diet History Questionnaire or Brief-type Diet History Questionnaire | 0 times/week |

**Supplementary Table 3.** Reference values of nutrients from the Dietary Reference Intakes for Japanese (2025) and prevalence of inadequate nutrient intake among young, middle-aged, and older women

| Reference value | | DRI  category | Prevalence of inadequate nutrient intake (%) | | |
| --- | --- | --- | --- | --- | --- |
|  |  |  | Young  (18–29 years) | Middle-aged  (30–64 years) | Older  (≥ 65 years) |
| **Nutrients with Estimated Average Requirement (EAR)** | | | | | |
| Protein | 40 g/day (≥ 18 years) | <EAR | 3 | 1 | 1 |
| Retinol | 450 µgRAE/day (18–29 years and ≥ 75 years);  500 µgRAE/day (30–74 years) | <EAR | 65 | 63 | 21 |
| Niacin | 9 mgNE/day (18–29 years and 50–74 years);  10 mgNE/day (30–49 years); 8 mgNE/day (≥ 75 years) | <EAR | 0 | 0 | 0 |
| Vitamin B_1_ | 0.6 mg/day (18–74 years); 0.5 mg/day (≥ 75 years) | <EAR | 19 | 10 | 7 |
| Vitamin B_2_ | 1 mg/day (18–74 years); 0.9 mg/day (≥ 65 years) | <EAR | 30 | 19 | 8 |
| Vitamin C | 80 mg/day (≥ 18 years) | <EAR | 60 | 55 | 13 |
| Vitamin B_6_ | 1 mg/day (≥ 18 years) | <EAR | 70 | 48 | 19 |
| Folate | 200 µg/day (≥ 18 years) | <EAR | 30 | 18 | 4 |
| Calcium | 550 mg/day (18–74 years); 500 mg/day (≥ 75 years) | <EAR | 83 | 76 | 44 |
| Iron | 9.3 mg/day (20–49 years); 5 mg/day (50–74 years); 4.5 mg/day (≥ 75 years) | <EAR | 96 | 70 | 3 |
| Magnesium | 230 mg/day (18–29 years); 240 mg/day (30–74 years); 220 mg/day (≥ 75 years) | <EAR | 83 | 66 | 40 |
| Zinc | 6 mg/day (18–29 years and ≥ 75 years); 6.5 mg/day (30–74 years) | <EAR | 18 | 27 | 13 |
| Copper | 0.6 mg/day (≥ 18 years) | <EAR | 0 | 0 | 0 |
| **Nutrients with Tentative Dietary Goal for preventing lifestyle-related diseases (DG)** | | | | | |
| Protein | 13–20% energy (18–49 years); 14–20% energy (50–64 years); 15–20% energy (≥ 65 years) | <DG | 51 | 41 | 27 |
|  |  | >DG | 0 | 0 | 16 |
| Fat | 20–30% energy (≥ 18 years) | <DG | 7 | 5 | 13 |
|  |  | >DG | 45 | 44 | 19 |
| Carbohydrate | 50–65% energy (≥ 18 years) | <DG | 18 | 25 | 20 |
|  |  | >DG | 11 | 6 | 10 |
| Fiber | 18 g/day (18–74 years); 17 mg/day (≥ 75 years) | <DG | 97 | 95 | 92 |
| Potassium | 2,600 mg/day (≥ 18 years) | <DG | 95 | 87 | 58 |
| Sodium (salt equivalent) | 6.5 g/day (≥ 18 years) | ≥DG | 87 | 93 | 97 |
| Saturated fatty acids | 7% energy (≥ 18 years) | >DG | 69 | 69 | 39 |

EAR, estimated average requirement; RAE, retinol activity equivalent; NE, niacin equivalent; DG, a tentative dietary goal for preventing lifestyle-related disease.

**Supplementary Table 4.** Percentiles of the Dietary Variety Score (DVS) and Alternative Dietary Variety Score (ADVS) among participants in each age group

|  |  | Percentiles of DVS and ADVS | | | | | | | | |
| --- | --- | --- | --- | --- | --- | --- | --- | --- | --- | --- |
|  |  | 1st | 5th | 10th | 25th | 50th | 75th | 90th | 95th | 99th |
| Young | DVS | 0 | 0 | 1 | 1 | 3 | 4 | 5 | 6 | 8 |
|  | ADVS | 0 | 0 | 0 | 1 | 2 | 4 | 5 | 6 | 7 |
| Middle-aged | DVS | 0 | 0 | 1 | 2 | 3 | 5 | 6 | 7 | 8 |
|  | ADVS | 0 | 0 | 1 | 2 | 3 | 4 | 6 | 6 | 8 |
| Older | DVS | 1 | 1 | 2 | 3 | 4 | 6 | 7 | 8 | 9 |
|  | ADVS | 1 | 1 | 2 | 3 | 4 | 6 | 7 | 7 | 9 |

DVS, Dietary Variety Score; ADVS, Alternative Dietary Variety Score

**Supplementary Table 5.** Percentage of participants consuming each food group at least once per day, overall and by Dietary Variety Score (DVS) category

|  | **All young (%)** | **DVS** | | | | | | | | | | |
| --- | --- | --- | --- | --- | --- | --- | --- | --- | --- | --- | --- | --- |
|  |  | **0** | **1** | **2** | **3** | **4** | **5** | **6** | **7** | **8** | **9** | **10** |
| Meat | 46 | 0 | 10 | 30 | 57 | 70 | 82 | 86 | 92 | 98 | 90 | 100 |
| Fish and shellfish | 14 | 0 | 1 | 4 | 7 | 20 | 31 | 48 | 68 | 70 | 100 | 100 |
| Soybeans and soybean products | 19 | 0 | 3 | 9 | 15 | 25 | 43 | 58 | 82 | 88 | 100 | 100 |
| Milk and dairy products | 27 | 0 | 12 | 19 | 26 | 36 | 48 | 62 | 61 | 70 | 90 | 100 |
| Green and yellow vegetables | 72 | 0 | 48 | 70 | 88 | 93 | 97 | 99 | 100 | 100 | 100 | 100 |
| Seaweeds | 11 | 0 | 2 | 4 | 7 | 12 | 24 | 40 | 51 | 78 | 80 | 100 |
| Potatoes | 3 | 0 | 0 | 0 | 1 | 4 | 3 | 9 | 17 | 48 | 60 | 100 |
| Fruits | 20 | 0 | 4 | 11 | 16 | 28 | 37 | 54 | 69 | 78 | 100 | 100 |
| Eggs | 24 | 0 | 5 | 15 | 20 | 36 | 51 | 57 | 66 | 80 | 80 | 100 |
| Fats and oils | 50 | 0 | 15 | 39 | 62 | 75 | 84 | 87 | 93 | 93 | 100 | 100 |
|  | **All middle-aged (%)** | **DVS** | | | | | | | | | | |
|  |  | **0** | **1** | **2** | **3** | **4** | **5** | **6** | **7** | **8** | **9** | **10** |
| Meat | 52 | 0 | 12 | 29 | 49 | 66 | 72 | 87 | 87 | 91 | 96 | 100 |
| Fish and Shellfish | 23 | 0 | 2 | 5 | 10 | 25 | 37 | 48 | 71 | 78 | 88 | 100 |
| Soybeans and soybean products | 34 | 0 | 3 | 13 | 26 | 38 | 51 | 67 | 85 | 94 | 92 | 100 |
| Milk and dairy products | 39 | 0 | 14 | 27 | 34 | 44 | 55 | 64 | 64 | 80 | 83 | 100 |
| Green and yellow vegetables | 82 | 0 | 49 | 74 | 89 | 95 | 97 | 100 | 100 | 100 | 100 | 100 |
| Seaweeds | 18 | 0 | 1 | 4 | 10 | 14 | 29 | 36 | 58 | 80 | 100 | 100 |
| Potatoes | 4 | 0 | 0 | 0 | 1 | 3 | 8 | 10 | 17 | 26 | 54 | 100 |
| Fruits | 29 | 0 | 2 | 13 | 22 | 29 | 44 | 57 | 66 | 89 | 96 | 100 |
| Eggs | 23 | 0 | 5 | 10 | 14 | 22 | 35 | 47 | 64 | 67 | 92 | 100 |
| Fats/oil | 50 | 0 | 11 | 26 | 46 | 64 | 71 | 84 | 88 | 94 | 100 | 100 |
|  | **All older (%)** | **DVS** | | | | | | | | | | |
|  |  | **0** | **1** | **2** | **3** | **4** | **5** | **6** | **7** | **8** | **9** | **10** |
| Meat | 31 | 0 | 0 | 2 | 14 | 21 | 35 | 49 | 66 | 77 | 83 | 100 |
| Fish and Shellfish | 60 | 0 | 3 | 23 | 42 | 63 | 73 | 84 | 90 | 95 | 97 | 100 |
| Soybeans and soybean products | 47 | 0 | 2 | 11 | 26 | 41 | 55 | 70 | 83 | 95 | 97 | 100 |
| Milk and dairy products | 49 | 0 | 7 | 26 | 37 | 42 | 59 | 63 | 72 | 86 | 90 | 100 |
| Green and yellow vegetables | 95 | 0 | 81 | 88 | 95 | 96 | 97 | 100 | 100 | 99 | 100 | 100 |
| Seaweeds | 20 | 0 | 0 | 2 | 6 | 13 | 21 | 29 | 51 | 58 | 76 | 100 |
| Potatoes | 11 | 0 | 0 | 3 | 1 | 6 | 8 | 15 | 26 | 36 | 79 | 100 |
| Fruits | 55 | 0 | 3 | 19 | 34 | 51 | 71 | 81 | 85 | 84 | 97 | 100 |
| Eggs | 25 | 0 | 1 | 5 | 11 | 21 | 24 | 35 | 48 | 79 | 86 | 100 |
| Fats/oil | 51 | 0 | 1 | 21 | 35 | 47 | 56 | 74 | 80 | 91 | 97 | 100 |

Values are percentage (%); DVS, Dietary Variety Score.

**Supplementary Table 6.** Percentage of participants consuming each food group at least once per day, overall and by Alternative Dietary Variety Score (ADVS) category

|  | **All young (%)** | **ADVS** | | | | | | | | | | |
| --- | --- | --- | --- | --- | --- | --- | --- | --- | --- | --- | --- | --- |
|  |  | **0** | **1** | **2** | **3** | **4** | **5** | **6** | **7** | **8** | **9** | **10** |
| Meat | 46 | 0 | 14 | 46 | 62 | 74 | 79 | 83 | 90 | 95 | 90 | 100 |
| Fish and shellfish | 14 | 0 | 2 | 5 | 13 | 24 | 44 | 51 | 66 | 77 | 100 | 100 |
| Soybeans and soybean products | 19 | 0 | 3 | 9 | 18 | 36 | 49 | 80 | 85 | 91 | 100 | 100 |
| Milk and dairy products | 27 | 0 | 11 | 21 | 32 | 45 | 54 | 62 | 66 | 82 | 100 | 100 |
| Green and yellow vegetables | 72 | 0 | 52 | 79 | 91 | 95 | 99 | 99 | 100 | 100 | 100 | 100 |
| Seaweeds | 11 | 0 | 1 | 4 | 9 | 21 | 32 | 41 | 71 | 68 | 90 | 100 |
| Potatoes | 3 | 0 | 0 | 0 | 2 | 4 | 7 | 13 | 31 | 45 | 70 | 100 |
| Fruits | 20 | 0 | 5 | 10 | 22 | 35 | 48 | 63 | 68 | 100 | 100 | 100 |
| Eggs | 24 | 0 | 7 | 15 | 32 | 42 | 52 | 64 | 73 | 73 | 90 | 100 |
| Whole grains | 16 | 0 | 5 | 11 | 19 | 24 | 34 | 43 | 51 | 68 | 60 | 100 |
|  | **All middle-aged (%)** | **ADVS** | | | | | | | | | | |
|  |  | **0** | **1** | **2** | **3** | **4** | **5** | **6** | **7** | **8** | **9** | **10** |
| Meat | 52 | 0 | 15 | 40 | 59 | 66 | 75 | 81 | 88 | 87 | 100 | 100 |
| Fish and shellfish | 23 | 0 | 2 | 5 | 16 | 29 | 46 | 59 | 69 | 80 | 100 | 100 |
| Soybeans and soybean products | 34 | 0 | 3 | 13 | 28 | 46 | 64 | 79 | 92 | 98 | 89 | 100 |
| Milk and dairy products | 39 | 0 | 16 | 24 | 41 | 51 | 59 | 66 | 73 | 91 | 78 | 100 |
| Green and yellow vegetables | 82 | 0 | 53 | 79 | 92 | 98 | 99 | 100 | 100 | 100 | 100 | 100 |
| Seaweeds | 18 | 0 | 1 | 5 | 10 | 21 | 33 | 50 | 72 | 85 | 89 | 100 |
| Potatoes | 4 | 0 | 0 | 0 | 2 | 5 | 8 | 13 | 24 | 37 | 78 | 100 |
| Fruits | 29 | 0 | 1 | 14 | 22 | 37 | 50 | 67 | 84 | 96 | 89 | 100 |
| Eggs | 23 | 0 | 5 | 10 | 18 | 29 | 42 | 51 | 61 | 72 | 89 | 100 |
| Whole grains | 15 | 0 | 5 | 9 | 12 | 19 | 23 | 35 | 37 | 54 | 89 | 100 |
|  | **All older (%)** | **ADVS** | | | | | | | | | | |
|  |  | **0** | **1** | **2** | **3** | **4** | **5** | **6** | **7** | **8** | **9** | **10** |
| Meat | 31 | 0 | 0 | 7 | 16 | 24 | 40 | 49 | 63 | 78 | 74 | 100 |
| Fish and shellfish | 60 | 0 | 4 | 29 | 42 | 65 | 77 | 83 | 93 | 89 | 100 | 100 |
| Soybeans and soybean products | 47 | 0 | 1 | 10 | 26 | 41 | 60 | 78 | 88 | 94 | 100 | 100 |
| Milk and dairy products | 49 | 0 | 4 | 20 | 40 | 48 | 59 | 65 | 77 | 91 | 91 | 100 |
| Green and yellow vegetables | 95 | 0 | 78 | 87 | 95 | 96 | 99 | 99 | 99 | 100 | 100 | 100 |
| Seaweeds | 20 | 0 | 0 | 1 | 7 | 12 | 21 | 36 | 55 | 69 | 83 | 100 |
| Potatoes | 11 | 0 | 0 | 1 | 3 | 5 | 9 | 18 | 33 | 41 | 78 | 100 |
| Fruits | 55 | 0 | 4 | 21 | 34 | 57 | 72 | 80 | 87 | 91 | 96 | 100 |
| Eggs | 25 | 0 | 0 | 7 | 12 | 20 | 27 | 42 | 54 | 74 | 87 | 100 |
| Whole grains | 34 | 0 | 9 | 16 | 24 | 32 | 37 | 48 | 51 | 74 | 91 | 100 |

Values are percentage (%); ADVS, Alternative Dietary Variety Score.
